# Mitochondria-related genes and metabolic profiles of innate and adaptive immune cells in primary Sjögren’s syndrome

**DOI:** 10.3389/fimmu.2023.1156774

**Published:** 2023-07-11

**Authors:** Danyang Luo, Lei Li, Yicheng Wu, Yi Yang, Yulin Ye, Jiawei Hu, Yiming Gao, Naiyan Zeng, Xiaochun Fei, Ning Li, Liting Jiang

**Affiliations:** ^1^ Department of Stomatology, Ruijin Hospital, Shanghai Jiao Tong University School of Medicine, College of Stomatology, Shanghai Jiao Tong University, Shanghai, China; ^2^ Department of Pathology, Ruijin Hospital, Shanghai Jiao Tong University School of Medicine, Shanghai, China; ^3^ Core Facility of Basic Medical Sciences, Shanghai Jiao Tong University School of Medicine, Shanghai, China; ^4^ Key Laboratory of Cell Differentiation and Apoptosis of Chinese Ministry of Education, Department of Pathophysiology, Shanghai Jiao Tong University School of Medicine, Shanghai, China

**Keywords:** Sjogren’s syndrome, mitochondria, mitochondrial metabolism, immune cell, transcriptomics, RNA sequencing

## Abstract

**Background:**

Primary Sjogren’s syndrome (pSS) is a prototypical systemic autoimmune disease characterised by lymphocyte infiltration and immune-complex deposition in multiple organs. The specific distribution of immune cell populations and their relationship with mitochondria remain unknown.

**Methods:**

Histological analysis was performed to assess the specific distribution of innate and adaptive immune cell populations in labial salivary gland (LSG) samples from 30 patients with pSS and 13 patients with non-pSS. The ultrastructural morphometric features of mitochondria within immune cells were observed under the transmission electron microscope (TEM). RNA sequencing was performed on LSG samples from 40 patients with pSS and 7 non-pSS patients. The Single-sample Gene Set Enrichment Analysis (ssGSEA), ESTIMATE, and CIBERSORT algorithms and Pearson correlation coefficients were used to examine the relationship between mitochondria-related genes and immune infiltration. Weighted Gene Co-expression Network Analysis (WGCNA) was used to identify the mitochondria-specific genes and the related pathways based on the immune cell types.

**Results:**

HE staining revealed a massive infiltration of plasma cells with abundant immunoglobulin protein distributed around phenotypically normal-appearing acinar and ductal tissues of patients with pSS. Immunohistochemical analyses revealed that innate immune cells (macrophages, eosinophils and NK cells) were distributed throughout the glandular tissue. Dominant adaptive immune cell infiltration composed of B cells, CD4^+^T cells and CD8^+^ T cells or ectopic lymphoid follicle-like structures were observed in the LSGs of patients with pSS. TEM validated the swelling of mitochondria with disorganised cristae in some lymphocytes that had invaded the glandular tissue. Subsequently, bioinformatic analysis revealed that innate and adaptive immune cells were associated with different mitochondrial metabolism pathways. Mitochondrial electron transport and respiratory chain complexes in the glandular microenvironment were positively correlated with innate immune cells, whereas amino acid and nucleic acid metabolism were negatively correlated with adaptive immune cells. In addition, mitochondrial biogenesis and mitochondrial apoptosis in the glandular microenvironment were closely associated with adaptive immune cells.

**Conclusion:**

Innate and adaptive immune cells have distinct distribution profiles in the salivary gland tissues of patients with pSS and are associated with different mitochondrial metabolic pathways, which may contribute to disease progression.

## Introduction

Primary Sjögren syndrome (pSS) is a systemic autoimmune disease characterised by dryness in the mouth and eyes, chronic pain and fatigue and several other extraglandular manifestations (1). The underlying pathological mechanism is complex and remains unclear. The histopathological features of pSS primarily manifest as lymphocytic infiltration and immune-complex deposition (2). Labial salivary gland (LSG) biopsy is usually used in the diagnosis of pSS, in which the focus score (FS) of ≥1 (i.e. ≥1 focus per 4 mm^2^) indicates a positive result (3, 4). The most characteristic feature of pSS is focal lymphocytic sialadenitis (FLS), which is used in the classification of pSS (3). FLS is characterised by infiltration of T and B lymphocytes. B cell activation-mediated clinical manifestations in the pathogenesis of pSS may increase the risk of B-cell lymphoma (5). Accumulation of B and T cells occurs in the advanced and early stages of pSS, respectively. Cytotoxic T cells are directly involved in the destruction of the LSG (6). Although activated CD4^+^T cells with T helper 1 (Th1) and Th2 phenotypes are present, Th17 and follicular helper T (Tfh) cells predominate and may provide a stimulus for B lymphocytes. In addition to the involvement of type I and type II interferons (IFNs) in the innate immune system, crosstalk exists between IL­21 (mainly secreted by Tfh cells) and B cell activating factor (BAFF) during B-cell activation (7).

Individual genetic susceptibility that is affected by multiple environmental factors can influence the development of pSS. Some autoantigens that are expressed by the epithelium of the exocrine glands, such as Ro/SSA and La/SSB, can induce a chronic inflammatory microenvironment that drives and activates the differentiation of immune cells (8). In the local inflammatory microenvironment of exocrine glands, activated immune cells further activate epithelial cells and recruit other immune cells, resulting in the continuous interaction between epithelial and immune cells (1, 8, 9). In response to viral infections, type I IFNs are produced by innate immune cells, including dendritic cells (DCs), to activate T and B cells that produce cytokines and autoantibodies to stimulate DCs to present more autoantigens in systemic autoimmune diseases (10). However, at present, the exact molecular mechanisms underlying the development of pSS remain unknown.

Mitochondria present in almost all cells are biosynthetic and signalling organelles that support cellular function. They are the metabolic hubs of the cell, regulating energy production by coordinating the electron transport chain (ETC) and tricarboxylic acid (TCA) cycle (11). Recent studies have indicated that mitochondria are necessary for the activation, proliferation and function of immune cells (12, 13), such as monocytes/macrophages (14), T/B cells (12, 15), and DCs (16). Alissafi et al. revealed that regulatory T cells (Tregs) undergo metabolic reprogramming with elevated mitochondrial oxidative stress and a robust DNA damage response during autoimmunity (17). Similarly, the mitochondrial respiratory chain is required for the Tregs suppression capacity (18). The metabolic functions of immune cells heavily rely on mitochondria. Abnormal mitochondrial metabolism of immune cells has been reported in autoimmune diseases, such as rheumatoid arthritis (RA) (19) and systemic lupus erythematosus (SLE) (20). Dysfunctional mitochondria released as components can act as pathogen-associated molecular patterns (PAMPs) and damage-associated molecular patterns (DAMPs), such as mtDNA and reactive oxygen species (ROS), to induce an inflammatory response—mainly in the innate immune system—by activating pattern recognition receptors (PRRs) in pSS (21, 22). In a previous study, we demonstrated the mitochondria-related phenotypic changes in the LSGs of patients with pSS (23). However, the role of mitochondrial dysfunction in immune cells in the pathogenesis of pSS remains unclear. Therefore, understanding the mechanisms underlying immune cell disorders is necessary.

This study aimed to examine the distribution of innate and adaptive immune cells, expression of mitochondrial functional proteins and ultrastructural changes in the mitochondria of immune cells in the LSGs of patients with pSS. Transcriptomic analysis revealed the possible mitochondria-related pathways associated with different immune cells. Additionally, changes in oxidative phosphorylation and the metabolic reprogramming of mitochondria in innate and adaptive immune cells were observed. Altogether, the findings of this study suggest a relationship between LSG and mitochondrial changes in immune cells, thus laying a foundation for further research into the mechanisms underlying the development of pSS.

## Materials and methods

### Patients and labial salivary gland biopsy

A total of 90 participants (70 patients with pSS and 20 age- and sex-matched non-pSS sicca controls) were included in this study. pSS was diagnosed according to the 2016 American College of Rheumatology/European League Against Rheumatism (ACR/EULAR) classification criteria (24). Individuals who presented with xerostomia or xerophthalmia but did not meet the ACR/EULAR classification criteria for pSS were classified as non-pSS. LSG samples and clinical information were collected after the patients had signed an informed consent form. No participant was receiving any immunosuppressive or steroid medication at the time of LSG biopsy. This study was reviewed and approved by the Ethics Committee of Ruijin Hospital, Shanghai Jiao Tong University School of Medicine and the Chinese Clinical Trial Registry (ChiCTR2000039820).

### Histological staining

The LSG samples were fixed in fresh 4% neutral formaldehyde overnight, placed into an automatic tissue dehydrator for dehydration, embedded in paraffin, and cut into 5-mm-thick serial sections for haematoxylin and eosin (HE) and immunohistochemical (IHC) staining. HE and IHC staining were performed according to the manufacturer’s instructions. For IHC staining, the LSG tissue sections were deparaffinised, rehydrated in a graded series of ethanol, subjected to antigen retrieval and blocked. Subsequently, the sections were incubated with primary antibodies (detailed information is listed in **Supplementary Table S1**) at 25°C for 20 minutes. The sections were washed with PBS; incubated with secondary antibodies; stained with 3,3’-diaminobenzidine (DAB, K5007, DAKO, Denmark) counterstained with haematoxylin. Thereafter, the sections were visualised using the BOND Polymer Refine Detection kit (DS9800, Leica Biosystems). The experiment was performed on a Leica Bond RX automated staining platform (Leica Biosystems).

### Transmission electron microscopy

The ultrastructure of LSGs was visualised *via* transmission electron microscopy (TEM) as described previously (23, 25). LSG tissue samples were fixed in 2.5% glutaraldehyde with 0.1-M phosphate-buffered saline (PBS, pH = 7.4), postfixed in 1% osmium tetroxide and dehydrated in a graded series of ethanol. The samples were embedded in a solution of 812 resin (EMS, TED PELLA, USA) and propylene oxide and incubated for 48 hours. The samples were transversely cut into sections of 70–90 nm using a diamond knife (EM UC7; Leica, Wetzlar, Germany) and stained with lead citrate. Images were obtained on a transmission electron microscope (H-7650; Hitachi, Japan).

### RNA sequencing and basic analysis

The LSGs samples of 40 patients with pSS and 7 patients with non-pSS were used for RNA-seq. Total RNA was extracted using the TRIzol reagent (Invitrogen, USA), quantified on an Agilent 2100 bioanalyser (Agilent Technologies, CA, USA) and a NanoDrop spectrophotometer (Thermo Fisher Scientific Inc.) and separated on 1% agarose gel. 1 µg of total RNA with RIN of >6.5 was used to create the library. The next-generation sequencing library was designed according to the manufacturer’s protocol. The prepared library was multiplexed and uploaded to the Illumina Novaseq system (Illumina, CA, USA). The sequence used a 2x-150-bp paired-end (PE) configuration. The HiSeq Control Software (HCS), OLB and GApipeline-1.6 (Illumina) management software were used for image analysis and identification. To eliminate technical errors, Cutadpt (V1.9.1) was used to process the data in the fastq file to obtain clean, high-quality data.

The limma and DEseq2 Bioconductor packages were used to identify differentially expressed genes (DEGs) based on the RNA-seq data (|logFC| > 0.5, p < 0.05). These DEGs were demonstrated on Venn diagrams and volcano plots. Interactive relationships and protein-protein interaction (PPI) networks of the overlapping DEGs were evaluated using the STRING database (http://string-db.org) (26). GO functional annotation and Kyoto Encyclopedia of Genes and Genomes (KEGG) pathway enrichment analyses were performed using the cluster profiler package in R (27). Based on the integrated GO and KEGG data, Metascape was used to identify all ontology terms that were significantly common among genes (28). Proteomaps (www.proteomaps.net) based on RNA transcript data were drawn to visualise three levels of functional categories based on KEGG pathway analysis (www.genome.jp/kegg/) (29)

### Analysis of immune cell infiltration and the glandular microenvironment

The cell type of each cluster (43 immune cells and 8 salivary glandular compositions) was identified using the CellMarker database and by aligning marker genes to known signature genes reported in previous studies (30). The Stromal, Immune and ESTIMATE scores of LSG samples were calculated using the ESTIMATE R package (http://bioinformatics.mdanderson.org/estimate/index.html).

The single-sample gene set enrichment analysis (ssGSEA) (31) and CIBERSORT (http://cibersort.stanford.edu) algorithms were used to estimate the proportion of innate and adaptive immune cell types in the LSGs of patients with pSS. The ComplexHeatmap (version 2.10.0) package in R (32) was used to construct heatmaps and perform cluster analysis. The ‘pheatmap’ package was used to examine the correlation (Spearman analysis) between the abundance of immune cells and the expression of hub genes.

### Weighted gene co-expression network analysis and gene set enrichment analysis

Weighted gene co-expression network analysis (WGCNA) was performed using the R package WGCNA to identify the module most relevant to immune infiltration (33, 34). Modules with interconnected features were screened based on network analysis, correlation coefficients and hierarchical clustering. To identify mitochondria-related genes highly correlated with innate or adaptive immune cells, the hub mitochondria-related genes were selected by intersecting genes in WGCNA modules and genes identified from the Mitocarta 3.0 database (35). GSEA was performed using the GSEA (version 2.0) software (31, 36). Curated gene sets for GSEA were obtained from http://software.broadinstitute.org/gsea/index.jsp. Enrichment was considered significant when the p-value was < 0.05. The correlation between hub immune cells and mitochondrial metabolic pathways was examined using the Mantel test and Pearson correlation coefficients in the pSS group and visualized using the ‘ggcor’ R package (version 0.9.8.1) (https://github.com/houyunhuang/ggcor) based on ‘ggplot2’ package.

### Statistical analysis

Statistical analysis was performed using Student’s t-test, one-way ANOVA or Chi-square test in the GraphPad Prism. Quantitative data were expressed as the mean ± standard deviation (SD). A p-value of < 0.05 was considered statistically significant.

## Results

### Distribution and proportion of innate and adaptive immune cells in the LSGs of patients with pSS

**Figure 1:**
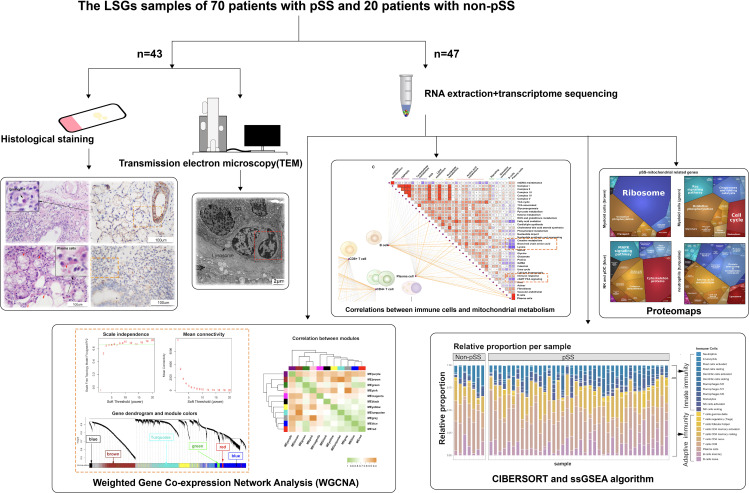
Schematic diagram of the workflow of histological and transcriptomic analysis.

A flowchart illustrating the overall study protocol is shown in **Figure 1**. A total of 70 patients with pSS and 20 sex- and age-matched Non-pSS patients were included. The clinical data of all participants were collected from their medical records (**Table 1**). Patients with FS of ≥ 1 and anti-SSA antibody-positive status were included in the pSS group, whereas those with FS of < 1 and anti-SSA antibody-negative status were included in the control group. Patients in the pSS group were found to have elevated serum IgG levels (17.98 ± 7.72 versus 12.94 ± 3.09 g/L, p < 0.01), ANA-positive status (94.3% versus 65%, p < 0.01), anti-Ro52-positive status (77.1% versus 45%, p < 0.01), and anti-SSB-positive status (41.4% versus 0, p < 0.001).

**Table 1:** Clinical data of patients with pSS and Non-pSS included in this study.

	pSS (n=70)	Non-pSS (n=20)
***Gender (Female)***	57/70	18/20
***Age***	52.04 ± 10.01	52.90 ± 9.14
***Chisholm Grade***	***3:*** 44	***0:*** 11
	***4:*** 26	***1:*** 4
	–	***2:*** 5
***ANA+ (%)***	94.3**	65
***Anti-SSA+ (%)***	100	0
***Anti-Ro52+(%)***	77.1**	45
***Anti-SSB+ (%)***	41.4***	0
***IgG (g/L)***	17.98 ± 7.72**	12.94 ± 3.09
***IgA (g/L)***	2.92 ± 1.22	2.43 ± 0.95
***IgM (g/L)***	1.43 ± 0.90	1.55 ± 0.92
***C3 (g/L)***	1.05 ± 0.28	1.12 ± 0.21
***C4 (g/L)***	0.25 ± 0.10	0.29 ± 0.14
***Hypocomplementemia***	6/70	2/18
***ESSDAI***	3.87 ± 1.12	0

Data are mean ± standard deviation (SD) or n (%). *p<0.05; **p<0.01; ***p<0.001.

ANA, anti-nucleic antibody. ESSDAI, EULAR Sjögren’s Syndrome Disease Activity Index.

**Figure 2:**
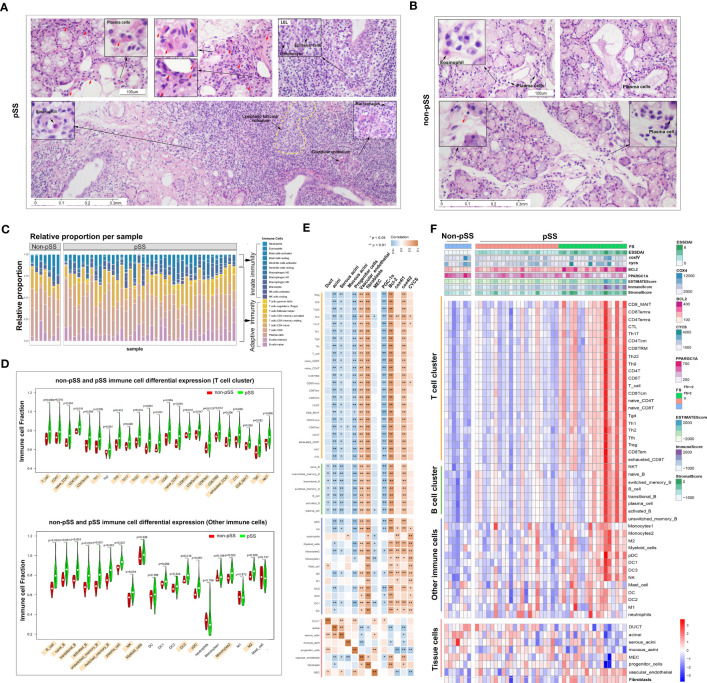
Distribution and proportion of innate and adaptive immune cells in the immune microenvironment of labial salivary glands (LSGs) of patients with primary Sjogren’s syndrome (pSS). **(A)** Histological features of focal lymphocytic sialadenitis (FLS) in the LSGs of patients with pSS, including lymphoepithelial lesions (LELs) and germinal centre (GC)-like structures. The red arrow represents plasma cells containing immunoglobulins (haematoxylin and eosin staining; original magnification at ×100 or ×400). **(B)** Various immune cells were scattered in the glandular tissue of patients without pSS (haematoxylin and eosin staining; original magnification at ×200 or ×400). **(C)** The proportion of innate and adaptive immune cells infiltrating the glandular tissue was determined using the CIBERSORT algorism. **(D)** Violin plot demonstrating the abundance of 43 types of immune cells evaluated using the ssGSEA algorithm. **(E)** Correlation between the expression of marker genes of the salivary gland and mitochondria and the abundance of immune cells in LSGs in the pSS and control groups. Orange represents a positive correlation, and blue represents a negative correlation. **(F)** Heatmap demonstrating the enrichment scores of the abovementioned genes and immune cells evaluated using the ssGSEA algorithm and stromal, immune and ESTIMATE scores calculated using the ESTIMATE algorithm. Each column represents an individual patient sample, and each row represents an individual gene or immune cell type, ordered *via* unsupervised hierarchical clustering.

HE staining was performed to visualise the distribution of immune cells in the LSGs of patients in both groups. In the pSS group, the structure of LSGs was destroyed, and excessive infiltration of immune cells was observed in LSGs (**Figure 2A**). A large number of plasma cells (red arrow) containing immunoglobulins was distributed around the acini and ducts. Similarly, the deposition of abnormal immunoglobulins was found in morphologically normal acini and ductal tissues. Some regions showed lymphoepithelial lesions (LELs) and germinal centre (GC)-like structures. According to morphological characteristics, a few eosinophils were scattered at the periphery of the lesions. Macrophages were mainly distributed in the residual glandular epithelial/myoepithelial regions. Conventionally, the glandular tissue of non-pSS patients had very small low levels of immune cell infiltration, and fewer plasma cells containing immunoglobulins (**Figure 2B**).

Salivary gland tissues subjected to RNA sequencing were analysed to quantify immune cell infiltration using CIBERSORT, ESTIMATE, and ssGSEA algorithms. CIBERSORT was used to calculate the proportion of 22 immune cell types, and the results showed that adaptive immune cells primarily infiltrated the LSGs of patients with pSS (**Figure 2C**). The results of ssGSEA showed that the proportion of infiltrating immune cells (both innate and adaptive) in LSGs was significantly higher in the pSS group than in the control group (p < 0.05) (**Figure 2D**). Furthermore, the relationship between the abundance of immune cells and expression of genes related to glandular tissue and mitochondria was examined (**Figures 2E, F**). The abundance of almost all immune cells was negatively correlated with the expression of marker genes of acini (mainly serous acini) and progenitor cells and the mitochondrial biogenesis-related gene PPARGC1A. However, it was positively correlated with the expression of marker genes of vascular endothelial cells and fibroblasts and mitochondria-related genes (BCL2 and COX4l2). The expression of marker genes of ducts and myoepithelial cells (MECs) was negatively correlated with the abundance of B cells and innate immune cells, respectively (**Figure 2E**). These results were verified in the pSS and control groups. Conventionally, the expression of BCL2 gradually increased and that of PPARGC1A decreased with the increase in FS. The ESTIMATE algorithm was used to assess the overall immune infiltration and stromal content (37). The results revealed that the pSS group had significantly higher Immune, Stromal, and ESTIMATE scores. These results were consistent with those of ssGSEA. In addition, the expression of the mitochondria-related genes cytochrome c oxidase (COX) -IV and cytochrome c (CYCS) was associated with the pathological features of pSS (**Figure 2F**). Altogether, these results indicate that the specific glandular microenvironment alters the proportion and distribution of immune cells and is associated with mitochondrial function.

### Histological analysis of innate immune cell infiltration in the LSGs of patients with pSS

**Figure 3:**
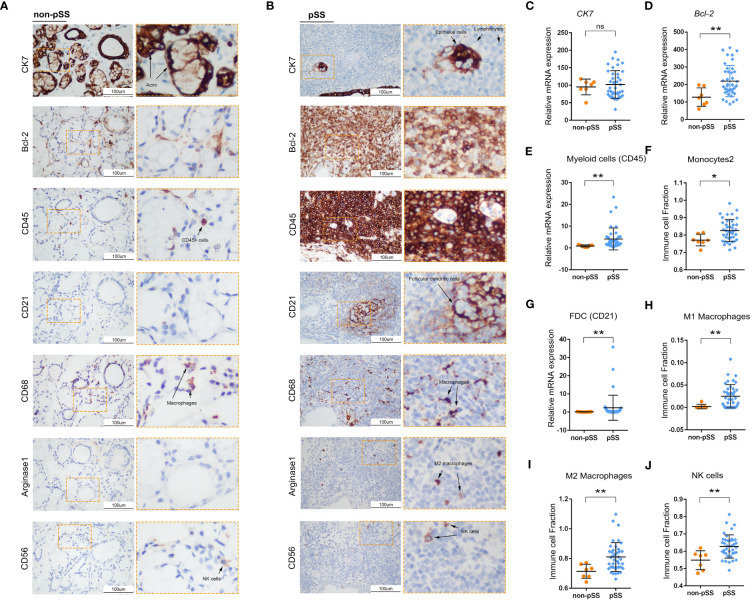
Histological analysis of innate immune cell infiltration in LSG tissues. **(A, B)** Representative images of IHC staining for CK7, Bcl-2, CD45, CD21, CD68, Arginase-1 and CD56 in human LSGs in the control **(A)** and pSS **(B)** groups (scale bar = 100 μm). **(C–E)** Statistical analysis of the expression of cytokeratin 7 (CK7) **(C)**, Bcl-2 **(D)** and CD45 **(E)** in human LSG tissues based on RNA-sequencing data (ns, no significant difference; *, p < 0.05; **, p < 0.01). **(F)** Monocyte infiltration and ssGSEA scores were compared between the two groups using the Mann–Whitney test (*, p < 0.05; **, p <0.01). **(G)** Statistical analysis of the expression of CD21 in human LSG tissues based on RNA-sequencing data. **(H–J)** Infiltration of M1 macrophages **(H)**, M2 macrophages **(I)** and NK cells **(J)** and ssGSEA scores were compared between the two groups using the Mann–Whitney test (*, p < 0.05; **, p < 0.01).

To characterise immune cells in the immune microenvironment of pSS, the cells were divided into innate and adaptive immune cell populations. The infiltration of innate immune cells was validated *via* IHC staining (**Figure 3**). A panel of innate immune cell-related markers was used, and ssGSEA was used to calculate infiltration scores based on RNA-seq data. Cytokeratin 7 (CK7, K7 or KRT7) is one of the marker genes of the salivary gland epithelium (38). Based on the analysis of CK7 expression, the LSG epithelium was found to be intact in the control group. However, almost no CK7 expression was observed in regions with infiltrated lymphocytes in patients with pSS. CK7 expression in the centre of FLS represented an epithelioid mass in regions with infiltrated lymphocytes. The antiapoptotic B-cell lymphoma 2 (BCL2) family members, including BCL2, can suppress autophagy and cell death and have been associated with ROS adaptation (39). BCL2 expression was high in not only acini and ducts in the non-pSS group but also regions with infiltrated lymphocytes in the pSS group. CD45 is expressed on all nucleated haematopoietic cells that can distinguish immune cells from salivary gland epithelial cells (40). Some epithelial tissue was observed in FLS, which was consistent with the results of CK7 staining analysis. CD21, a marker of follicular dendritic cells (FDCs), represents the GC structure (3). Arginase-1 is a marker of M2 polarisation, whereas CD68 represents the total macrophages (41). CD56 is known as a phenotypical marker of natural killer (NK) cells (42). FDCs, macrophages and NK cells were observed in the LSGs of pSS patients. FDCs were expressed in the GC, whereas macrophages, mainly M2 macrophages, and NK cells were distributed around the infiltrating foci. The expression of the marker genes of innate immune cells all showed an upward trend in the pSS group based on the RNA-seq (**Figure S1**). The results of histological analysis were consistent with those of RNA-seq, suggesting that the RNA-seq data were reliable.

### Expression patterns of mitochondria-related genes in innate immune cells

**Figure 4:**
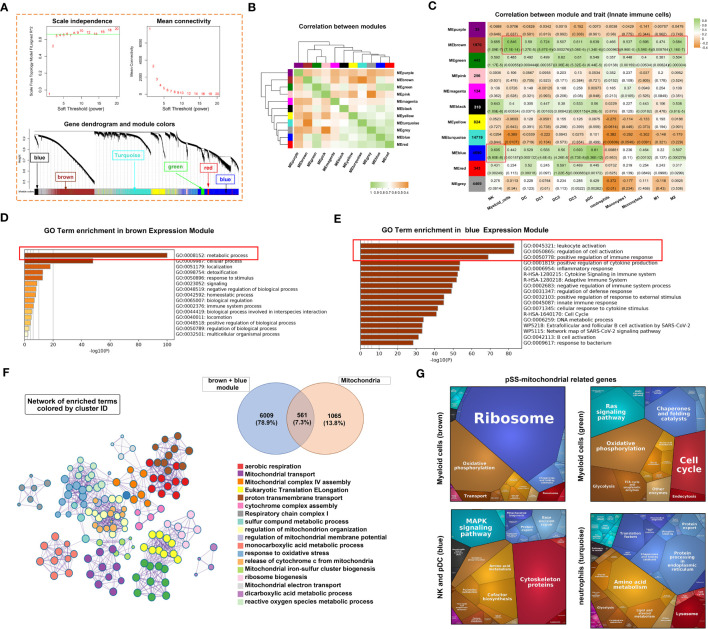
Innate immune-related co-expression network generated using WGCNA and mitochondrial-related gene enrichment analysis. **(A)** Analysis of the scale-free fit index (upper left panel) and the mean connectivity for various soft-thresholding powers (upper right panel). Different colours represent different gene modules (lower panel). **(B)** Hierarchical clustering correlation among WGCNA modules. A total of 11 gene co-expression modules were identified. **(C)** Heatmap demonstrating module–trait relationships. Green indicates a positive correlation and orange indicates a negative correlation between the expression of genes in the modules and the abundance of innate immune cells. The number in each immune cell indicates the correlation coefficient and p-value. **(D, E)** Bar graph demonstrating the results of GO functional annotation analysis performed using Metascape in the brown **(D)** and blue **(E)** modules. **(F)** Significantly enriched clusters marked in different colours in the network were annotated using Metascape (left panel). Different colours represent different GO terms. The Venn diagram (right panel) demonstrates the 561 overlapping genes between mitochondria-related genes and genes in the brown and blue modules. **(G)** Heatmap demonstrating functional categories in three innate immune cell types (different modules) as identified using Proteomaps (www.proteomaps.net). Each protein encoded by a known ID gene is represented by a polygon, and functionally related proteins are arranged in common and similar regions.

The ssGSEA scores of 12 innate immune cell types were used as phenotypic data for the WGCNA analysis to identify co-expressed genes and examine the correlation between gene modules and phenotypes. A total of 11 modules were displayed in different colours (**Figure 4A**). The correlation between different modules is presented in **Figure 4B**. Furthermore, the correlation between genes in these modules and marker genes of innate immune cells was examined (**Figure 4C**). The expression of genes in four modules, including brown, green, black and blue modules, was significantly positively correlated with the abundance of almost all types of innate immune cells. The turquoise module contained most genes, and the expression of these genes was significantly negatively correlated with only three cell types, including myeloid cells, neutrophils, and type 2 monocytes.

Metascape was used to identify signalling pathways associated with genes in the brown and blue modules. Based on the integrated results of GO and KEGG analyses, metabolic processes were mainly associated with genes in the brown module (**Figure 4D**). However, leukocyte activation, regulation of activation and positive regulation of immune response were associated with genes in the blue module (**Figure 4E**). Subsequently, the genes of the two modules were merged and intersected with mitochondria-related genes to obtain 561 genes as shown in the Venn diagram in **Figure 4F**. Based on these 561 genes, a hub term enrichment network was constructed using Metascape. Respiratory chain-related signalling pathways were at the core of the enrichment network. Furthermore, we selected four modules with a significant correlation and intersected genes in these modules with mitochondria-related genes. KEGG analysis of the overlapping genes revealed that pathways associated with ribosomes and oxidative phosphorylation were dominant in the brown module corresponding to myeloid cells (**Figure 4G**). Similarly, pathways associated with oxidative phosphorylation and Ras signalling were dominant in the green module corresponding to myeloid cells, those associated with cytoskeleton proteins and MAPK signalling were dominant in the blue module corresponding to NK cells and pDCs and those associated with amino acid, lipid and steroid metabolism were dominant in the turquoise module corresponding to neutrophils.

**Figure 5:**
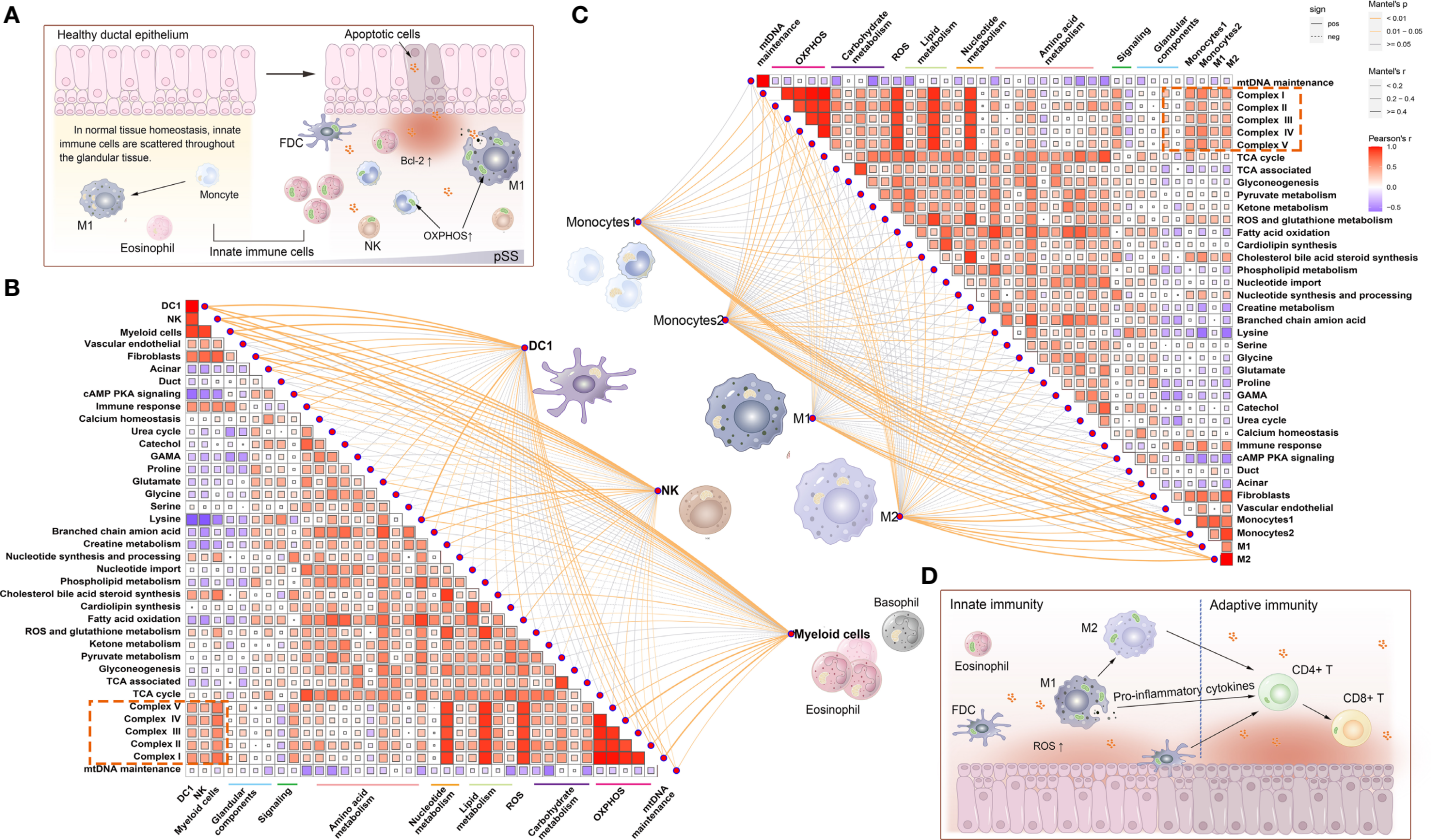
Correlation between mitochondrial metabolic pathways and innate immune cells. **(A)** Schematic representation of the distribution of innate immune cells under normal versus pathological conditions. During the development of pSS, apoptotic glandular epithelial cells release pro-inflammatory factors and ROS. ROS triggers mitochondrial apoptosis by regulating Bcl-2 expression, and scattered innate immune cells (macrophages, NK cells and neutrophils) are attracted to the inflamed sites. To meet the energy demands associated with inflammatory responses, these innate immune cells may have different energy metabolism mechanisms (such as mitochondrial OXPHOS) to produce ATP. **(B)** Correlation between the abundance of innate immune cells, namely, DC1, NK cells and myeloid cells, and the activity of mitochondrial metabolic pathways. **(C)** Correlation between the abundance of innate immune cells, namely, type 1 and 2 monocytes and M1 and M2 macrophages, and the activity of mitochondrial metabolic pathways. Statistical analysis was performed using the Mantel test, with the yellow line indicating p < 0.01 and the light yellow line indicating 0.01 < p < 0.05. The edge width corresponds to the r-value (Mantel test), and the edge colour indicates statistical significance. The relationship between variables was examined by estimating Pearson correlation coefficients. **(D)** Schematic diagram demonstrating that innate immune cells (such as macrophages and dendritic cells [DCs]) not only initiate immune responses but also simultaneously transfer the signal to lymphocytes and activate the adaptive immune response.

Owing to the high enrichment of metabolic processes (**Figure 4D**), we investigated the potential correlation among innate immune cells, glandular tissue and mitochondrial metabolism in the pSS group. The Mantel test was used for statistical analysis, and pattern diagrams were drawn (**Figure 5**). The abundance of innate immune cells (DC1, NK cells, myeloid cells, type 1 and 2 monocytes and M1 and M2 macrophages) was strongly positively correlated with the mitochondrial respiratory chain complex, fibroblast abundance, immune response, lipid metabolism and nucleotide synthesis. However, it was significantly negatively correlated with acinar cell abundance, cAMP–PKA signalling and amino acid metabolism (**Figures 5B, C**). Altogether, these results suggest that mitochondria-related metabolic pathways in innate immune cell populations are altered in patients with pSS.

### Adaptive immune cells were mainly distributed in the central region of the FLS in the LSGs of patients with pSS

**Figure 6:**
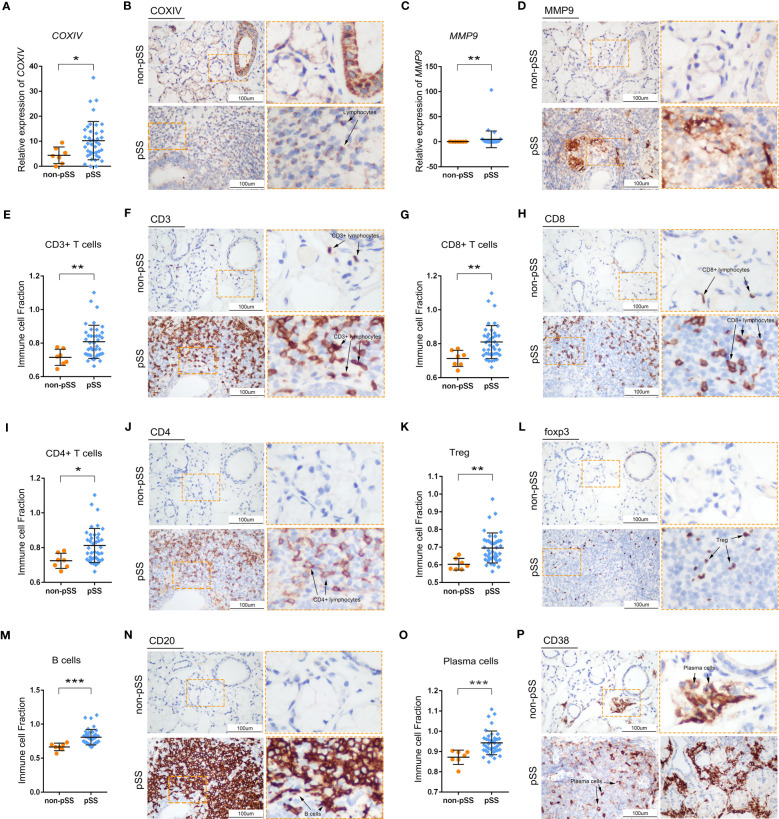
Histological analysis of adaptive immune cells in LSG tissues. **(A, B)** Statistical analysis of COX-IV expression based on RNA-sequencing data **(A)** and representative images of IHC staining for COX-IV **(B)** in human LSGs in the control and pSS groups (scale bar = 100 μm) (*, p < 0.05). **(C–D)** Statistical analysis of MMP9 expression based on RNA-sequencing data **(C)** and representative images of IHC staining for MMP9 **(D)** in human LSGs in the control and pSS groups (scale bar = 100 μm) (**, p < 0.01). **(E, F)** Statistical analysis of CD3 immune infiltration and ssGSEA scores based on RNA-sequencing data **(E)** and representative images of IHC staining for CD3 **(F)** in human LSGs in the control and pSS groups (scale bar = 100 μm) (**, p < 0.01). **(G, H)** Statistical analysis for CD8 immune infiltration and ssGSEA scores based on RNA-sequencing data **(G)** and representative images of IHC staining for CD8 **(H)** in human LSGs in the control and pSS groups (scale bar = 100 μm) (**, p < 0.01). **(I, J)** Statistical analysis of CD4 immune infiltration and ssGSEA scores based on RNA-sequencing data **(I)** and representative images of IHC staining for CD4 **(J)** in human LSGs in the control and pSS groups (scale bar = 100 μm) (*, p < 0.05). **(K, L)** Statistical analysis of Treg infiltration and ssGSEA scores based on RNA-sequencing data **(K)** and representative images of IHC staining for Foxp3 **(L)** in human LSGs in the control and pSS groups (scale bar = 100 μm) (**, p < 0.01). **(M, N)** Statistical analysis of B-cell infiltration and ssGSEA scores based on RNA-sequencing data **(M)** and representative images of IHC staining for CD20 **(N)** in human LSGs in the control and pSS groups (scale bar = 100 μm) (***, p < 0.001). **(O, P)** Statistical analysis of plasma cell infiltration and ssGSEA scores based on RNA-sequencing data **(O)** and representative images of IHC staining for CD38 **(P)** in human LSGs in the control and pSS groups (scale bar = 100 μm) (***, p < 0.001).

The distribution of adaptive immune cells was assessed *via* IHC staining. The expression of COX-IV, a subunit of COX translocation protons, was high not only in ductal epithelial cells in the control group but also in the immune cells of lymphoid infiltrating lesions in the pSS group (**Figure 6B**). Matrix metalloproteinase 9 (MMP9) is a family of endopeptidases that regulate extracellular matrix remodelling and contribute to the development of fibrosis (43). In the LSGs of patients with pSS, MMPs were expressed in clumps in the centre of lesions (**Figure 6D**). Additionally, CD3 (T cells), CD4 (T cells) and CD20 (B cells) were mainly expressed in the centre of FLS and constituted the main part of the GC structure (**Figures 6F, J, N**). CD8 (T cells), Foxp3 (Tregs) and CD38 (plasma cells) were scattered in the periphery of GC (**Figures 6H, L, P**). The expression of marker genes in adaptive immune cells is shown in **Figure S2**. The expression of CD3D (T cells) and MS4A1 (B cells) was low in the control group, whereas the distribution of CD38 (plasma cella) was consistent with the results of HE staining (**Figures 6O, P**, **2B**). These cell distribution trends were consistent with those observed in RNA-seq analysis and ssGSEA scores (**Figures 6A, C, E, G, I, K, M**, **S2**).

**Figure 7:**
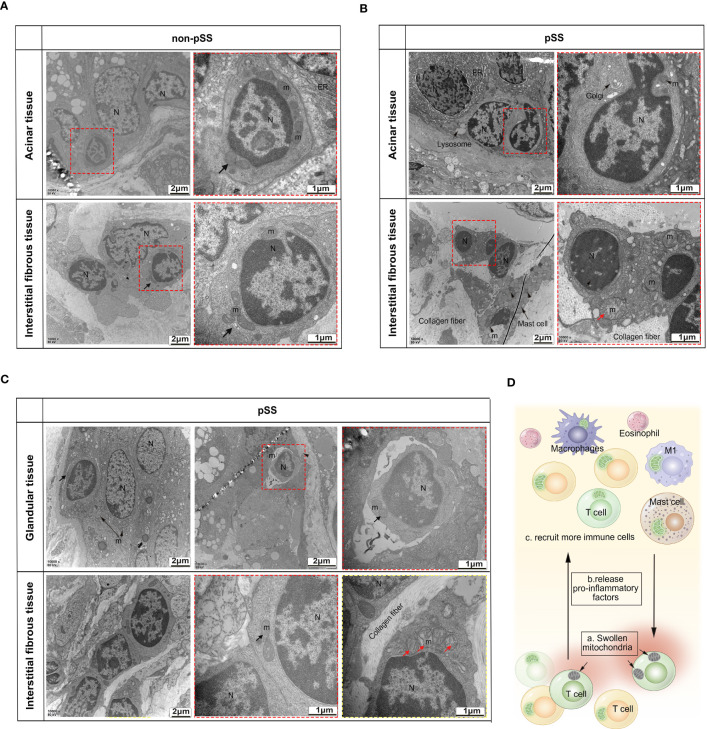
Transmission electron microscopic analysis of the ultrastructure of mitochondria in the immune cells of LSGs. **(A)** Representative TEM images demonstrating low levels of lymphocyte infiltration in the normal glandular tissue and interstitium and normal morphology of mitochondria and nuclei in lymphocytes. **(B, C)** TEM images validated that the number of abnormal mitochondria was increased in some tissue-invading lymphocytes (swollen mitochondria and intact nuclei membrane). **(D)** Schematic diagram demonstrating the accumulation of dysfunctional swollen mitochondria in some lymphocytes. Apoptotic lymphocytes may increase inflammation in the glandular microenvironment, thereby recruiting more immune cells.

As mitochondrial function is closely associated with mitochondrial morphology, dysfunctional mitochondria are swollen and fragmented, whereas healthy mitochondria are thin and long. TEM was used to observe the ultrastructure of mitochondria in the immune cells of LSGs. As shown in **Figure 7A**, lymphocyte infiltration was very low in normal glandular tissues and interstitium. Healthy lymphocytes have less cytoplasm, normal mitochondrial morphology and clear mitochondrial cristae. In the pSS group, more lymphocytes were found to invade the basement membrane of the acini, ducts and interstitium. Although the surrounding acinar cells exhibited endoplasmic reticulum stress and karyopyknosis, the invading lymphocytes retained the morphological features of mitochondrial cristae (**Figure 7B**). However, lymphocytes with swollen mitochondria and broken cristae and lysosomes were seen in the interstitial fibrous tissue (**Figures 7B, C**). These changes did not occur in all lymphocytes but were only partially visible (**Figure 7D**). Although ductal cells contained massively swollen mitochondria, some invading lymphocytes had normal mitochondria.

### Patterns of mitochondria-related metabolism in adaptive immune cells were similar to those in innate immune cell

**Figure 8:**
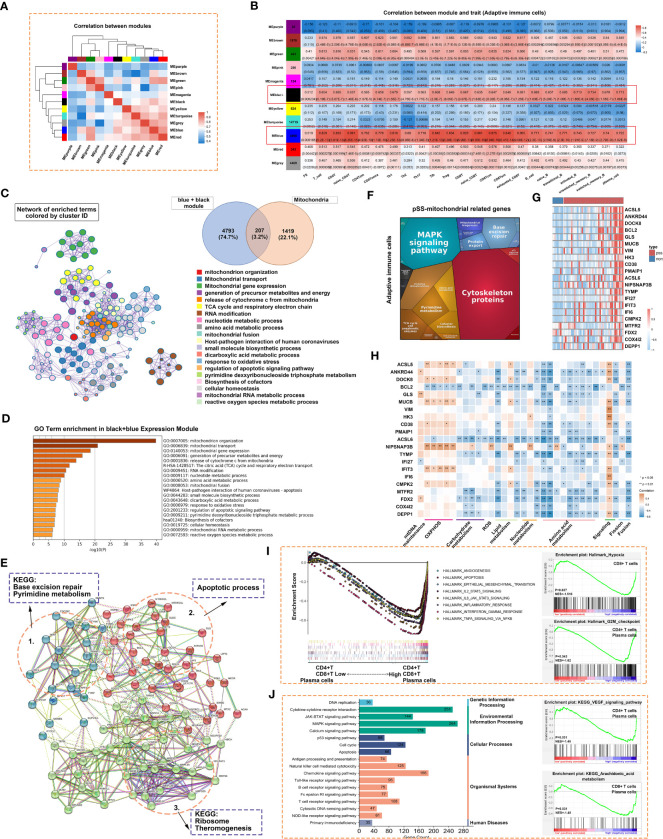
Adaptive immune-related co-expression network generated using WGCNA and mitochondrial-related gene enrichment analysis. **(A)** Hierarchical clustering correlation among WGCNA modules. A total of 11 gene co-expression modules were identified. **(B)** Heatmap demonstrating module–trait relationships. Green indicates a positive correlation and orange indicates a negative correlation between the expression of genes in the modules and the abundance of innate immune cells. The number in each immune cell indicates the correlation coefficient and p-value. **(C)** Significantly enriched clusters marked in different colours in the network were annotated using Metascape (left panel). Different colours represent different GO terms. The Venn diagram (right panel) demonstrates the 207 overlapping genes between mitochondria-related genes and genes in the black and blue modules. **(D)** Bar graph demonstrating the results of GO functional annotation analysis of the 207 genes performed using Metascape. **(E)** PPI network revealed three clusters related to pyrimidine metabolism, apoptosis and ribosomes. **(F)** Heatmap demonstrating the functional categories of adaptive immune cells based on the 207 mitochondria-related genes as examined using Proteomaps. Each protein encoded by a known ID gene is represented by a polygon, and functionally related proteins are arranged in common and similar regions. **(G)** Heatmap of 21 differentially expressed genes related to mitochondria. **(H)** The correlation between the 21 genes and mitochondrial metabolic pathways was examined *via* Pearson correlation analysis. Orange indicates a positive correlation, whereas blue indicates a negative correlation (*, p < 0.05; **, p < 0.01). **(I)** GSEA was used to evaluate the abundance of CD4^+^ T, CD8^+^ T and plasma cells in the Hallmark gene set. **(J)** GSEA was used to evaluate the abundance of CD4^+^ T, CD8^+^ T and plasmas in the KEGG gene set.

The ssGSEA scores of 22 types of T and B cells and FS were selected as phenotypic data for WGCNA to identify co-expressed genes and examine the inter-relationship among gene modules, pathological features and phenotypes (**Figures 8A, B**). The expression of genes in the blue and black modules had the strongest correlation with the abundance of adaptive immune cell populations. Genes in these two modules were integrated and intersected with mitochondria-related genes to obtain 207 genes. Metascape was used to construct the corresponding enrichment term networks for these overlapping genes, and the results are shown in the Venn diagram in **Figure 8C**. The term ‘release of cytochrome c from mitochondria’ was at the centre of the network (**Figure 8C**), whereas the term ‘mitochondrion organization’ had the highest enrichment score (**Figure 8D**).

Cytoscape was used to construct a PPI network of 207 genes with more than 5 nodes. As shown in **Figure 8E**, genes related to base excision repair and pyrimidine metabolism, apoptotic process, and ribosome and thermogenesis formed the three main cores of the network. Proteomaps were constructed to visualise mitochondria-related signalling pathways in the blue and black modules identified *via* KEGG analysis (**Figure 8F**). Pathways associated with cytoskeleton proteins and MAPK signalling accounted for the largest proportion. DEGs were identified based on the RNA-seq data. The DEGs and the 207 genes in the PPI network were intersected to obtain 21 overlapping genes. These 21 genes were highly expressed in the pSS group, especially in the Chisholm-grade 4 subgroup (**Figure 8G**). Furthermore, correlation analysis revealed that the expression of all genes, except for BCL2 and ACSL6, was positively correlated with oxidative phosphorylation and negatively correlated with mitochondrial metabolome and fusion (**Figure 8H**). ssGSEA was used to analyse relevant signalling pathways enriched in CD4^+^ T, CD8^+^ T and plasma cells based on Hallmark and KEGG gene sets. Apoptosis and the JAK–STAT signalling pathway were shared by the three immune cell types in both gene sets (**Figures 8I, J**). In particular, hypoxia was unique to CD8^+^ T cells and the G2-M checkpoint was shared by CD4^+^ T and plasma cells in the Hallmark gene set (**Figure 8I**). The VEGF signalling pathway was shared by CD4^+^ T and plasma cells and arachidonic acid metabolism was shared by CD8^+^ T and plasma cells in the KEGG gene set (**Figure 8J**).

**Figure 9:**
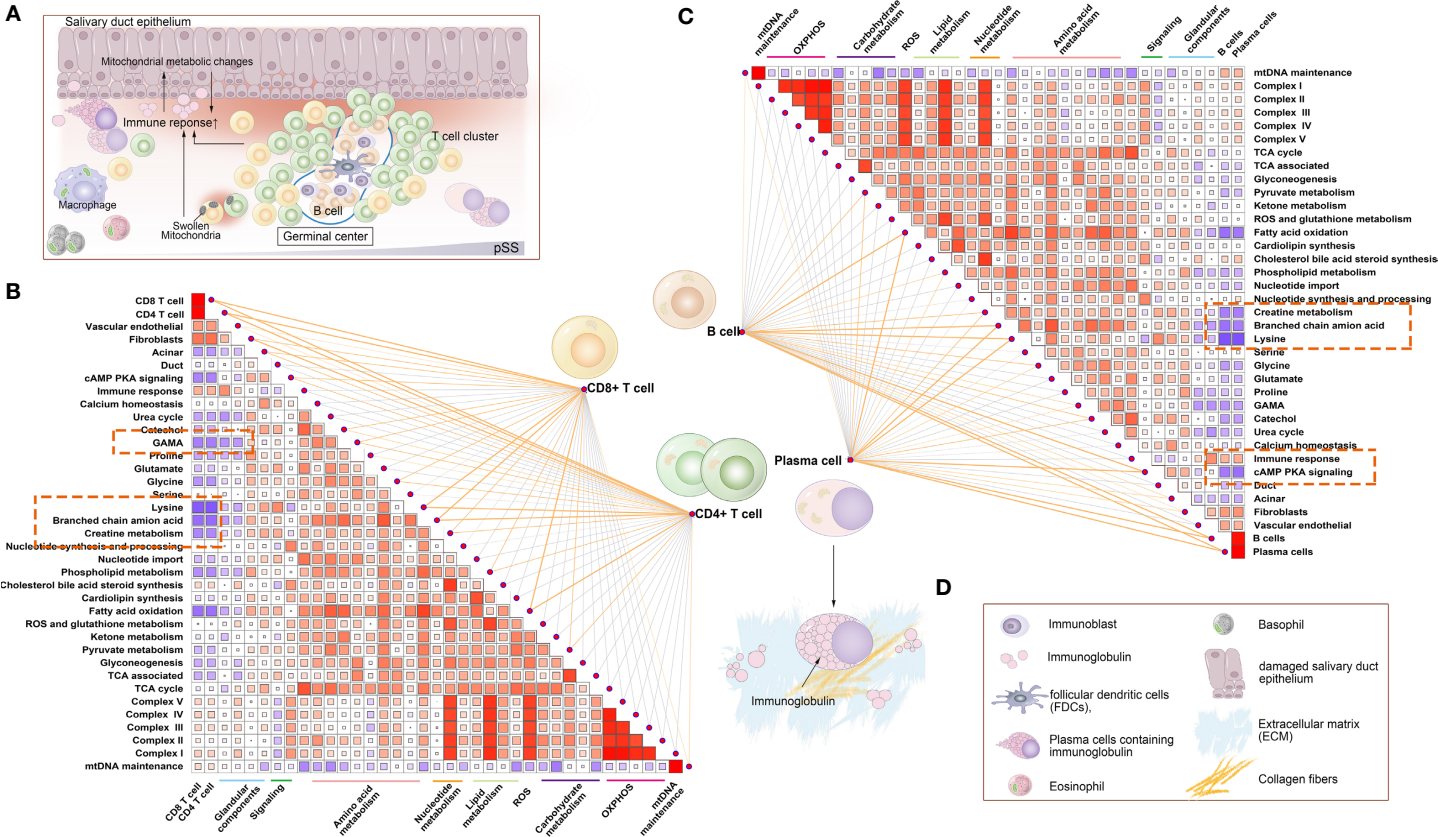
Correlation between mitochondrial metabolic pathways and adaptive immune cells. **(A)** Schematic diagram demonstrating typical lymphocytic foci in the LSG, a feature of pSS. The glandular microenvironment undergoes metabolic reprogramming when triggered by the immune system, which may be a result of an inflammatory environment and may be directly triggered by mitochondrial dysfunction in some immune cells. **(B)** Correlation between the abundance of CD4^+^ T and CD8^+^ T cells and mitochondrial metabolic pathways. **(C)** Correlation between B and plasma cells and mitochondrial metabolic pathways. Statistical analysis was performed using the Mantel test, with the yellow line indicating p < 0.01 and the light yellow line indicating 0.01 < p < 0.05. The edge width corresponds to the r-value (Mantel’s test), and the edge colour indicated statistical significance. The relationship between variables was examined by estimating Pearson correlation coefficients. **(D)** Inset demonstrating a schematic diagram of each element involved in **Figure 9A**.

The correlation between the abundance of CD4^+^ T, CD8^+^ T, B and plasma cells and the expression of mitochondria-related genes was examined (**Figure 9**). Similar to innate immune cells, these adaptive immune cells were negatively correlated with mitochondrial metabolism and positively correlated with immune responses and respiratory chain complexes.

## Discussion

This study demonstrated the infiltration characteristics of innate and adaptive immune cells in the LSGs of patients with pSS and the ultrastructure of mitochondria in the two types of immune cells. Mitochondria-related metabolic pathways in innate and adaptive immune cells were analysed using RNA-seq data. Abnormal mitochondria in salivary gland epithelial cells may act as DAMPs to trigger the formation of the local immune microenvironment (23). Patients with pSS had a higher positive rate of the ANA, anti-Ro52 and anti-SSB and higher levels of serum IgG.

pSS is one of the most prevalent systemic autoimmune diseases that is characterised by a triad of manifestations: dryness, pain and fatigue (44). Hydroxychloroquine, a common immunosuppressant, did not improve symptoms during 24 weeks of treatment of pSS (45). More clinical studies are needed to support the efficacy and safety of targeted T-cell drugs, such as Abatacept (46). Targeted B-cell agents, such as anti-B cell activating factor (BAFF) Belimumab (47) and Ianalumab (48), anti-CD20 Rituximab (47, 49), can relieve some systemic symptoms and remain the most promising therapeutic drugs for pSS (44). Therefore, new therapeutic targets targeting immune cells and the mechanisms underlying the development of pSS warrant further investigation. Mesenchymal stem cells (MSCs) treatment may provide a new therapy for pSS, since MSC treatment directed T cells toward Treg and Th2 and suppressed Th17 and Tfh responses (50).

The salivary gland epithelium is composed of acinar cells, ductal cells, MECs and progenitor cells. We have previously demonstrated that serous and mucous acini appear atrophied and the mucus content in LSGs is decreased in pSS, which may be some of the reasons for dryness in the mouth (23, 25). Salivary epithelial cells play a central role in regulating autoimmune responses by expressing various immune molecules, including human leukocyte antigen (HLA) class I molecules, CD40 (a key molecule in antigen presentation), FAS receptor (apoptosis-related molecule) and pro­inflammatory cytokines and chemokines (8). When salivary gland epithelial cells undergo apoptosis, the release of proinflammatory factors and ROS can induce the aggregation of innate immune cells. These innate immune cells are recruited to the injured LSG epithelial tissue, and the activity of respiratory chain complexes and anti-apoptosis function are enhanced to exert immune effects. Mitochondria in salivary glandular epithelial cells show an abnormal structure with increased lymphocyte infiltration. A few studies have focused on the role of mitochondrial metabolites (21), such as cytochrome c (51), mtDNA (52) or ROS, in the immune microenvironment after salivary gland epithelial injury in pSS. In this study, TEM revealed mitochondrial swelling and autophagosome accumulation in scattered lymphocytes and ductal cells of the LSGs of patients with pSS, which is an important finding. Numerous studies (15, 53, 54) have shown that the swollen state of mitochondria is related to the abnormal ion channels on the mitochondrial membrane, mainly calcium channels, and the dysregulation of ROS. Correlation analysis showed that metabolic pathways related to mitochondrial swelling, such as calcium homeostasis, ROS and glutathione metabolism, did not show correlation with immune cells. Therefore, we speculate that abnormal mitochondrial function in glandular tissue leads to apoptosis and induces immune cell aggregation. Although most lymphocytes continue to function normally in pSS, damaged mitochondria in immune cells may recruit more immune cells to infiltrate, causing progressive destruction of the LSG.

Glycolysis and oxidative phosphorylation are primary sources of cellular energy. Increased oxidative phosphorylation activity often indicates cell activation and a change in function. In this study, respiratory chain complexes and oxidative phosphorylation were identified as the link between innate immune cell populations and mitochondria. Innate immune cells exhibited strong oxidative respiratory activity. Respiratory chain complexes are extremely important for the differentiation of immune cells. Blocking complex I or III can inhibit the differentiation of naïve CD4^+^ T cells into effector CD4^+^ T cells (55). Complex II is critical for oxidative phosphorylation and the TCA cycle. Defects in complex II can sensitise intestinal epithelial cells to T-cell-mediated cytotoxicity, which further induces T-cell-mediated diseases (56).

Many innate immune cells, such as monocytes and M1 macrophages, produce ATP through glycolysis instead of the TCA cycle (13). M2 macrophages mainly rely on oxidative phosphorylation to perform their immune function, whereas M1 macrophages rely on glycolysis (14). In this study, both types of cells eventually exhibited higher levels of oxidative phosphorylation. M2 macrophages were less distributed in the LSGs that were dominated by M1 macrophages in patients with pSS. These results are consistent with those of IHC analysis. In a study, single-cell RNA sequencing revealed that the expression of genes in the oxidative phosphorylation module was high in mitotic GC B cells (57). Another study reported that Treg cells impaired mitochondrial oxidative phosphorylation during an autoimmune process (17). In this study, the abundance of immune cells was not strongly associated with the expression of TCA-related genes but was strongly positively correlated with the activity of the respiratory chain complex. This finding indicates that activation of these immune cells does not rely on the TCA cycle for energy. Furthermore, ROS metabolism was positively correlated with the abundance of immune cells. Mitochondrial ROS play an important role in intracellular signal transduction and immune regulation; however, excessive ROS production may damage cells and lead to immune regulation disorders (58). For example, a reduction in ROS levels can significantly decrease the antigen-presenting ability of pDCs (59). Therefore, alterations in oxidative phosphorylation and ROS-related signalling pathways are important features of immune cells in patients with pSS. The mitochondrial metabolic function of immune cells in the local immune microenvironment changes to exert effects on the immune response. Abnormal metabolic profiles can further recruit more immune cells, resulting in a vicious cycle. Therefore, maintaining mitochondrial function and intervening with metabolism may serve as promising strategies for the treatment of pSS in the future.

Peroxisome proliferator-activated receptor-γ (PPARγ) coactivator 1α (PGC-1α) is the main regulatory component of mitochondrial biogenesis and energetic adaptation (60). PGC-1α is an important mitochondria-related protein for the executive function of immune cells. The differentiation of human monocytes into DCs is accompanied by increased expression of PGC-1α (16). The expression of PGC-1α can support the formation of CD8^+^ T central memory (Tcm) cells to maintain metabolic stability and exert immune effects (61). However, in this study, the abundance of immune cells was negatively correlated with the expression of the PPARGC1A gene. We hypothesise that mitochondrial biogenesis is disrupted in dominant glandular tissue cells owing to immune cell infiltration, resulting in decreased expression of PPARGC1A. The potential role of PGC-1α in pSS warrants further investigation.

Several reviews have summarised the relationship between immune cells and mitochondria and their metabolic function in SLE (62) and ageing (63, 64). Another important finding of this study is that the activity of mitochondria-related metabolic pathways is altered in patients with pSS. Owing to the limitations of bulk RNA sequencing technology, only overall fluctuations in the glandular immune microenvironment and mitochondria-related metabolic pathways were observed. The specific metabolic characteristics of different cell groups should be examined using more advanced technology, such as single-cell RNA sequencing and spatial transcriptome sequencing. In addition, there is a certain proportion of healthy populations and patients with organ specific autoimmune diseases show anti-Ro52 positive in the current study. In order to avoid this bias, more control cases should be included in our future studies.

## Conclusion

This study suggests that innate and adaptive immune cells have distinct distribution profiles in the salivary glandular tissues of patients with pSS and highlights mitochondrial metabolic pathways that may contribute to disease progression. In response to inflammation in the glandular epithelium, some immune cells with normal mitochondria infiltrate the gland, whereas others with dysfunctional swollen mitochondria may increase inflammation in the glandular microenvironment and hence recruit more immune cells, exacerbating tissue destruction.

## Data availability statement

The datasets presented in this study can be found in online repositories. The names of the repository/repositories and accession number(s) can be found below: OER378348 (https://www.biosino.org/node/run/detail/OER378348).

## Ethics statement

The studies involving human participants were reviewed and approved by the ethics committee of Ruijin Hospital, Shanghai Jiao Tong University School of Medicine. The patients/participants provided their written informed consent to participate in this study.

## Author contributions

LJ and NL designed the overall research strategy. LJ and DL wrote the manuscript. LL, YW, YYang, YYe, and JH performed the experiments. YG, NZ and XF analyzed the data. All authors contributed to the article and approved the submitted version.
