# Supplementary material for: Mitochondria-related genes and metabolic profiles of innate and adaptive immune cells in primary Sjögren’s syndrome
Source: Front Immunol. 2023 Jul 11;14:1156774. doi: 10.3389/fimmu.2023.1156774 (PMC10366690; doi:10.3389/fimmu.2023.1156774)
Supplement: Supplementary file 1 [file DataSheet_1.docx]

**SUPPLEMENTARY FIGURES**

**
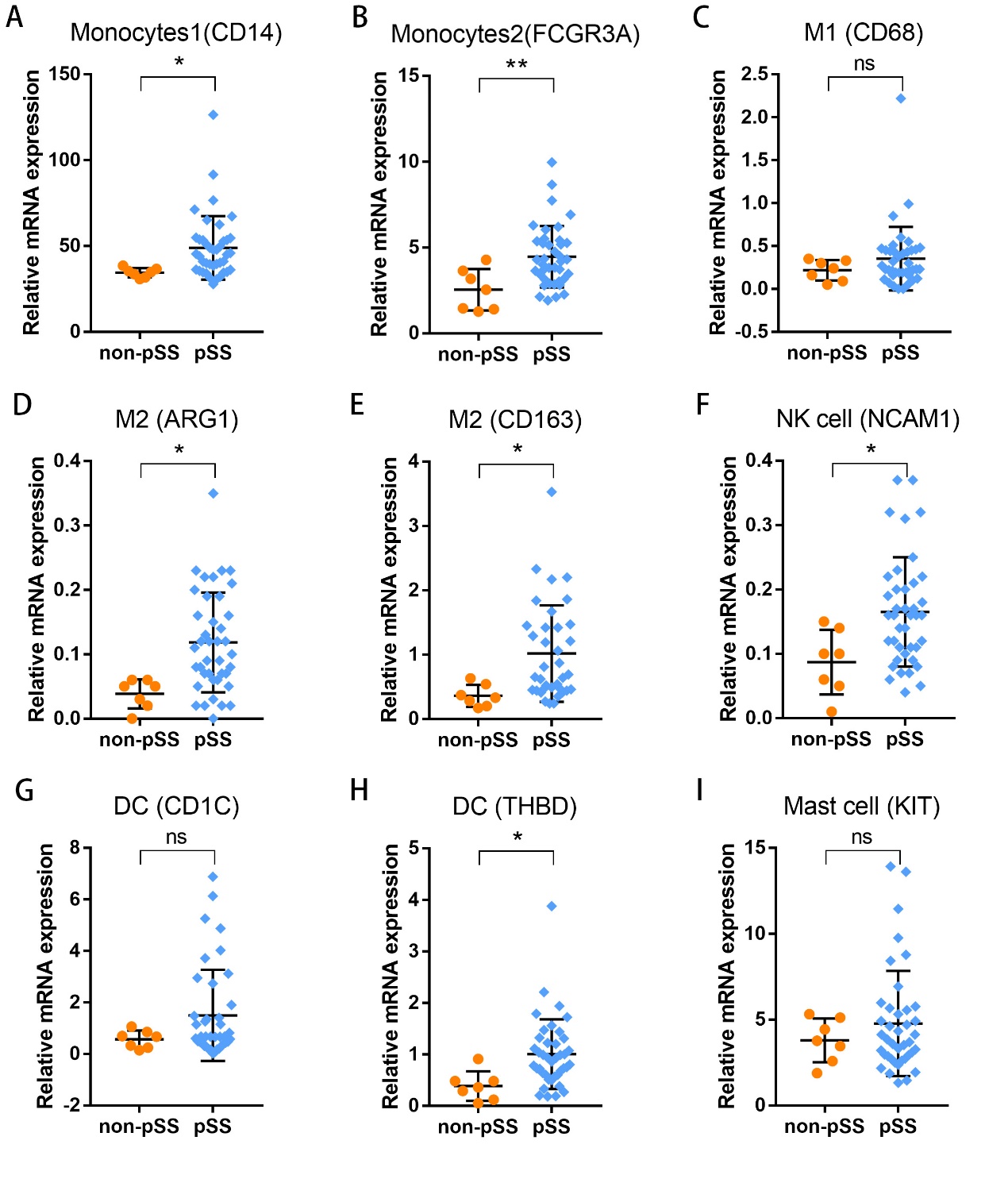
**

**Figure S1.** The expression of marker genes in innate immune cells. Statistic analysis for CD14 (Monocyte1) **(A)**, FCGR3A (CD16, Monocyte2) **(B)**, CD68 (Macrophage 1, M1) **(C)**, ARG1/CD163 (M2) **(D-E)**, CD56/NCAM1 (NK) **(F)**, CD1C and CD141 (DC) **(G-H)**, and KIT (mast cell) **(I)** genes expression from RNA sequencing results.


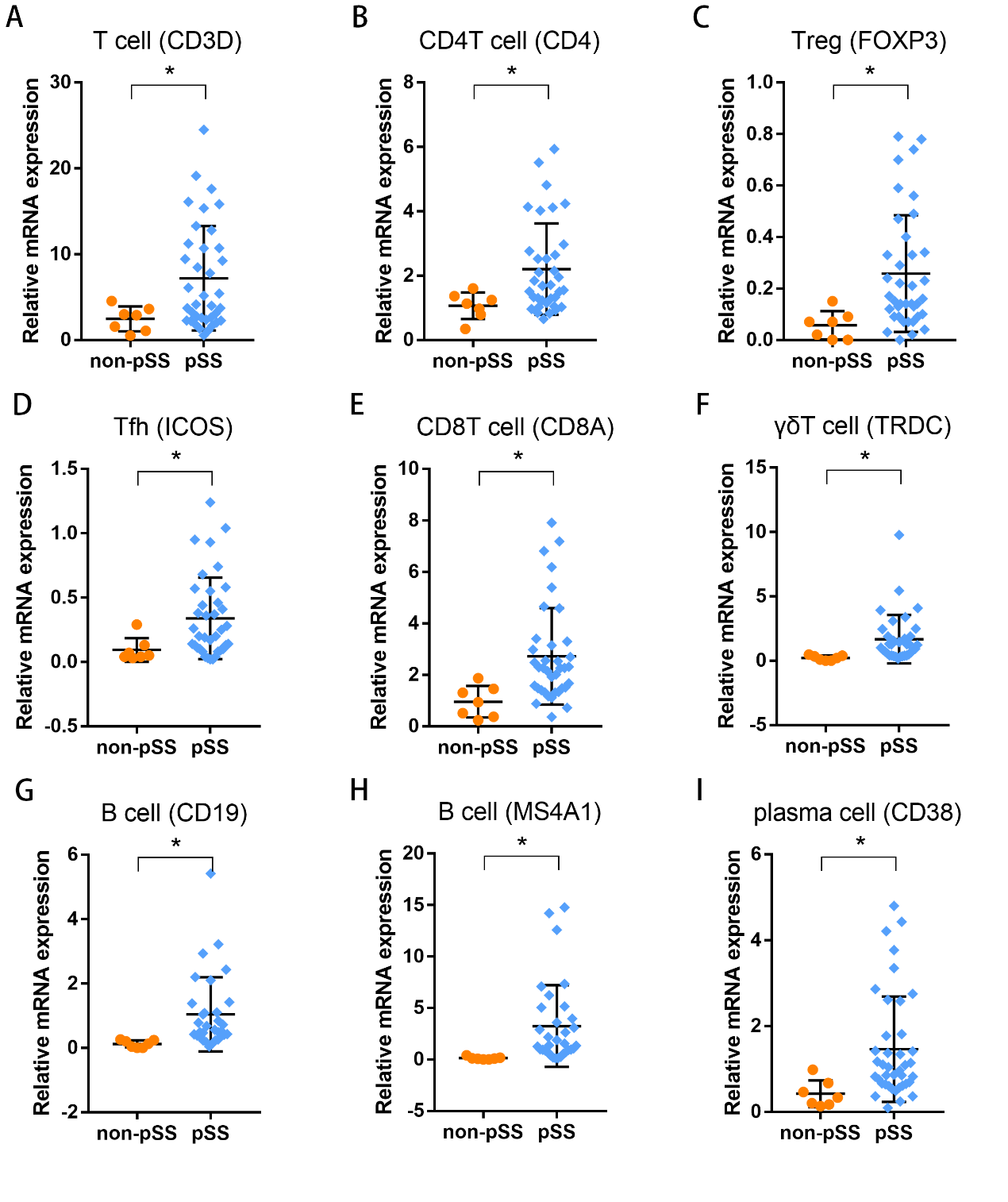


**Figure S2.** The expression of marker genes in adaptive immune cells. Statistic analysis for CD3D (T cell) **(A)**, CD4 (CD4^+^T cell) **(B)**, FOXP3 (Treg) **(C)**, ICOS (Tfh) **(D)**, CD8A (CD8^+^T cell) **(E)**, TRDC (γδT cell) **(F)**, CD19 and MS4A1 (B cell) **(G-H)**, and CD38 (plasma cell) **(I)** genes expression from RNA sequencing results.

**SUPPLEMENTARY TABLE**

Table S1 Primary antibodies detailed information

| antigen | dilution/concentration | Cat. # | | Company and Nation | |
| --- | --- | --- | --- | --- | --- |
| Cytokeratin 7 | undiluted | GA619 | Dako, Denmark | |  |
| Bcl-2 | undiluted | IR614 | Dako, Denmark | |  |
| CD45 | undiluted | GA751 | Dako, Denmark | |  |
| CD21 | 1:100 | ZL223 | Leica, Germany | |  |
| CD68 | undiluted | GA613 | Dako, Denmark | |  |
| CD56 | 1:100 | IR628 | Dako, Denmark | |  |
| Arginase 1 | 1:100 | ab133543 | Abcam, UK | |  |
| COX-IV | 1:1200 | ab202554 | Abcam, UK | |  |
| MMP9 | 1:100 | 10375-2-AP | Proteintech, USA | |  |
| CD3 | undiluted | GA503 | Dako, Denmark | |  |
| CD4 | undiluted | IR649 | Dako, Denmark | |  |
| CD8 | 1:100 | ab20034 | Abcam, UK | |  |
| Foxp3 | 1:100 | ab253297 | Abcam, UK | |  |
| CD20 | undiluted | GA604 | Dako, Denmark | |  |
| CD38 | undiluted | ZM-0422 | Zhongshan, China | |  |
